# The salinity tolerant poplar database (STPD): a comprehensive database for studying tree salt-tolerant adaption and poplar genomics

**DOI:** 10.1186/s12864-015-1414-7

**Published:** 2015-03-17

**Authors:** Yazhen Ma, Ting Xu, Dongshi Wan, Tao Ma, Sheng Shi, Jianquan Liu, Quanjun Hu

**Affiliations:** Molecular Ecology Group, State Key Laboratory of Grassland and Agro-Ecosystems, School of Life Sciences, Lanzhou University, Lanzhou, 730000 Gansu China

**Keywords:** Salt-tolerant, *Populus*, Genome database, Tree adaptation, Abiotic stress

## Abstract

**Background:**

Soil salinity is a significant factor that impairs plant growth and agricultural productivity, and numerous efforts are underway to enhance salt tolerance of economically important plants. *Populus* species are widely cultivated for diverse uses. Especially, they grow in different habitats, from salty soil to mesophytic environment, and are therefore used as a model genus for elucidating physiological and molecular mechanisms of stress tolerance in woody plants.

**Description:**

The Salinity Tolerant Poplar Database (STPD) is an integrative database for salt-tolerant poplar genome biology. Currently the STPD contains *Populus euphratica* genome and its related genetic resources. *P. euphratica*, with a preference of the salty habitats, has become a valuable genetic resource for the exploitation of tolerance characteristics in trees. This database contains curated data including genomic sequence, genes and gene functional information, non-coding RNA sequences, transposable elements, simple sequence repeats and single nucleotide polymorphisms information of *P. euphratica*, gene expression data between *P. euphratica* and *Populus tomentosa,* and whole-genome alignments between *Populus trichocarpa*, *P. euphratica* and *Salix suchowensis*. The STPD provides useful searching and data mining tools, including GBrowse genome browser, BLAST servers and genome alignments viewer, which can be used to browse genome regions, identify similar sequences and visualize genome alignments. Datasets within the STPD can also be downloaded to perform local searches.

**Conclusions:**

A new Salinity Tolerant Poplar Database has been developed to assist studies of salt tolerance in trees and poplar genomics. The database will be continuously updated to incorporate new genome-wide data of related poplar species. This database will serve as an infrastructure for researches on the molecular function of genes, comparative genomics, and evolution in closely related species as well as promote advances in molecular breeding within *Populus*. The STPD can be accessed at http://me.lzu.edu.cn/stpd/.

## Background

Salinity is a main environmental constraint that renders fields unproductive. It is also one of the most severe abiotic stress factors affecting plant growth and agricultural production worldwide [1]. To cope with this intractable problem, many researches have been undertaken to explore the physiological and molecular mechanisms of plants that naturally display high salt resistance or use plant breeding and biotechnological approaches to enhance the stress resistance of salt-sensitive plant species, especially those with significant economic importance [2-5].

The genus *Populus* is widely distributed and consists of many species that play important parts in bio-energy production, environmental protection and afforestation on degraded soils [6]. In addition to their conspicuous economic values, these woody species also exhibit different degrees of stress resistance as a consequence of adaptation to different habitats [7,8], thus being very suitable to address tree-specific questions of salt stress tolerance [9].

*Populus euphratica* Oliv. is a salinity tolerant poplar, which occurs in semiarid and arid areas [10]. It grows under unfavorable conditions such as saline soils, but sustains higher photosynthetic and growth rates than other poplar species under high salinity [3,11]. With the extraordinary adaptation to salt stress, it has become a model for elucidating salt resistance mechanisms in trees [12]. Breeders have tried to increase tree salt tolerance by crossing *P. euphratica* with other economical species. However, successful hybridization with positive features is scarce [7]. Therefore, molecular breeding provides a promising alternative.

Based on the recently completed whole genome sequence of the *P. euphratica* [12], we have built a comprehensive web-based database, STPD (http://me.lzu.edu.cn/stpd/), to facilitate researches on salinity tolerance and molecular breeding of *Populus*.

## Construction and content

**Figure 1:**
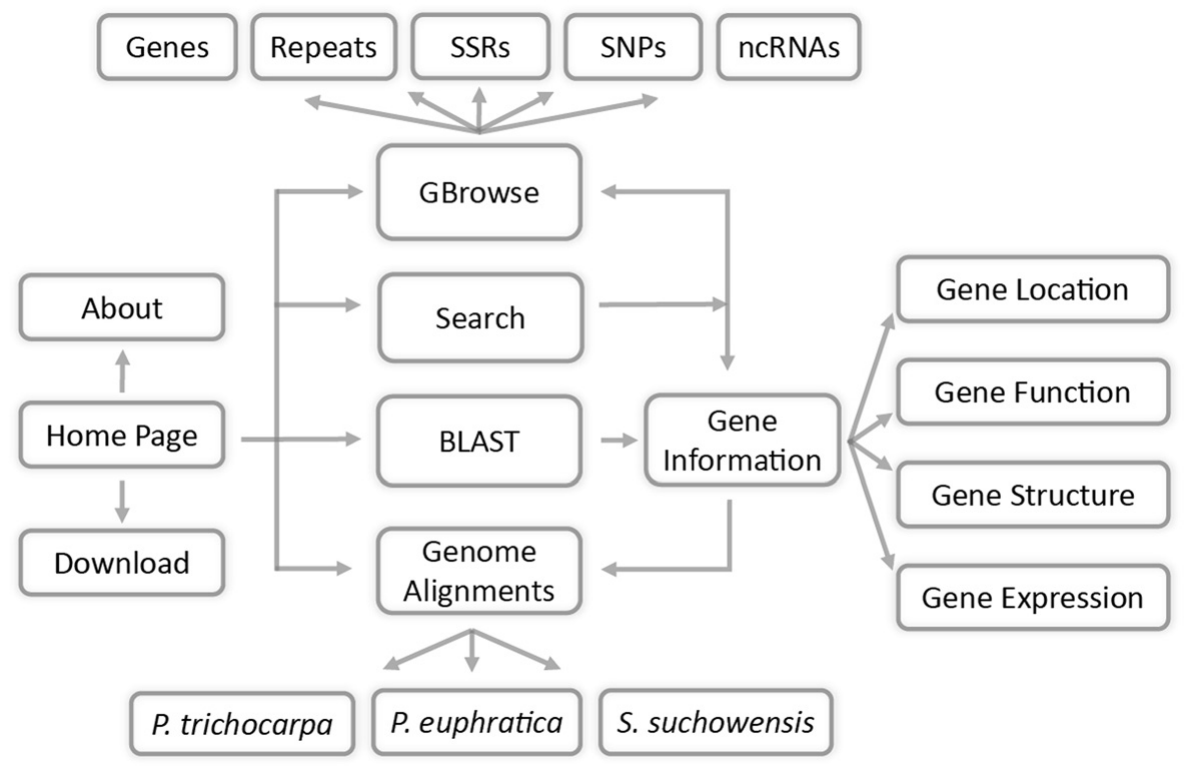
**Schematic overview of the STPD sitemap.** STPD includes Gene search, Gene information, Genome browser, Sequence search, Genome comparison, and Data downloading pages. Arrows are used to indicate flow of information.

The STPD currently gives public access to *P. euphratica* genome assembly version 1.1, which was sequenced and assembled using the fosmid pooling and hierarchical approach. The final assembly covers a total length of 496.5 Mb [12], and 34,279 protein-coding genes were predicted in the whole genome. In addition, 764 transfer RNAs, 706 ribosomal RNAs, 4,826 small nuclear RNAs and 266 microRNAs that supported by small RNA sequencing data were identified and included in the database. We also incorporated gene expression data, which is based on time-course profiling of differentially expressed genes between *P. euphratica* and *Populus tomentosa* (a salt-sensitive poplar) in response to salt stress. Moreover, a total of 18,938 universal pairs of simple sequence repeat (SSRs) primers were identified in the syntenic regions of *P. euphratica* and *Populus trichocarpa*, and these SSRs can be converted into genetic markers across most poplar species. The STPD includes GBrowse genome browser, gene search function, BLAST sequence searching and other intuitive tools to facilitate the analysis of the genetic data in salt-tolerant *Populus* (Figure 1).

### Genome component

Repeated sequences within the *P. euphratica* genome were identified by RepeatMasker (http://www.repeatmasker.org) with two different libraries. The first one is Repbase TE library (http://www.girinst.org/repbase) while the second one was produced by RepeatModeler, which yielded classification information for each repeat family and consensus sequences as a repeat library.

### Gene component

We obtained the gene dataset of *P. euphratica* using a variety of strategies, including RNA-seq, homology and *ab initio* gene prediction. The total predicted gene number is 34,279. Among these, 20038 (58.46%) genes are supported by RNA-seq and 32182 (93.88%) have a homologue either in *Ricinus communis*, *Cucumis sativus*, *P. trichocarpa* or *Prunus persica* [12]. This combined gene dataset was then used as a reference and has been integrated into the STPD. The genes were annotated with a variety of databases including Swiss-Prot/TrEMBL (http://www.uniprot.org), KEGG (http://www.genome.jp/kegg), InterPro (http://www.ebi.ac.uk/interpro) and Gene Ontology (http://geneontology.org). Swiss-Prot and TrEMBL annotations for the predicted proteins were generated by performing BLASTP searching (E-value ≤10^−5^) against the Swiss-Prot and TrEMBL databases. Genes were mapped to KEGG pathway using KAAS [13]. We also used InterProScan to annotate domains and motifs by comparing the genes to the public Pfam, PRINTS, PROSITE, ProDom and SMART, using HMMPfam, FPrintScan, ScanRegExp, ProfileScan, BlastProDom and HMMsmart with the following parameters: −format raw -goterms -iprlookup [14]. Gene Ontology information was then extracted from the InterProScan results with in-house Perl scripts.

### Comparative genomics

Willows (*Salix*) share a common ‘Salicoid’ whole-genome duplication events with poplars, and their chromosomal structures are highly similar [15]. To better understand the salt-tolerant adaptive evolution, we performed a whole-genome comparison between *P. euphratica*, *Salix Suchowensis* and *P. trichocarpa,* a salt-sensitive model plant with a high-quality reference genome [16], using Mercator and MAVID [17].

### Gene expression

The STPD also includes gene expression data of *P. euphratica* calluses in response to salt stress together with those of *P. tomentosa* (a salt-sensitive poplar). The analysis was conducted on salt-stressed and non-stressed samples (200 mM NaCl for 6, 12, 24 and 48 h), which generated from the same calluses used in genome sequencing [12].

### Population genomics

Whole-genome sequence data from four *P. euphratica* individuals located in China were integrated into the STPD. We aligned the reads to reference genome with Burrows-Wheeler Aligner (BWA) [18], filtered duplicated reads using samtools [19], and realigned INDELs with GATK [20]. Finally we called single nucleotide polymorphisms (SNPs) with samtools mpileup, and dropped low quality SNPs (Quality <30). In total, 5,894,808 SNPs were identified. The average alignment coverage is 9X per individual.

## Utility

### Gene search

**Figure 2:**
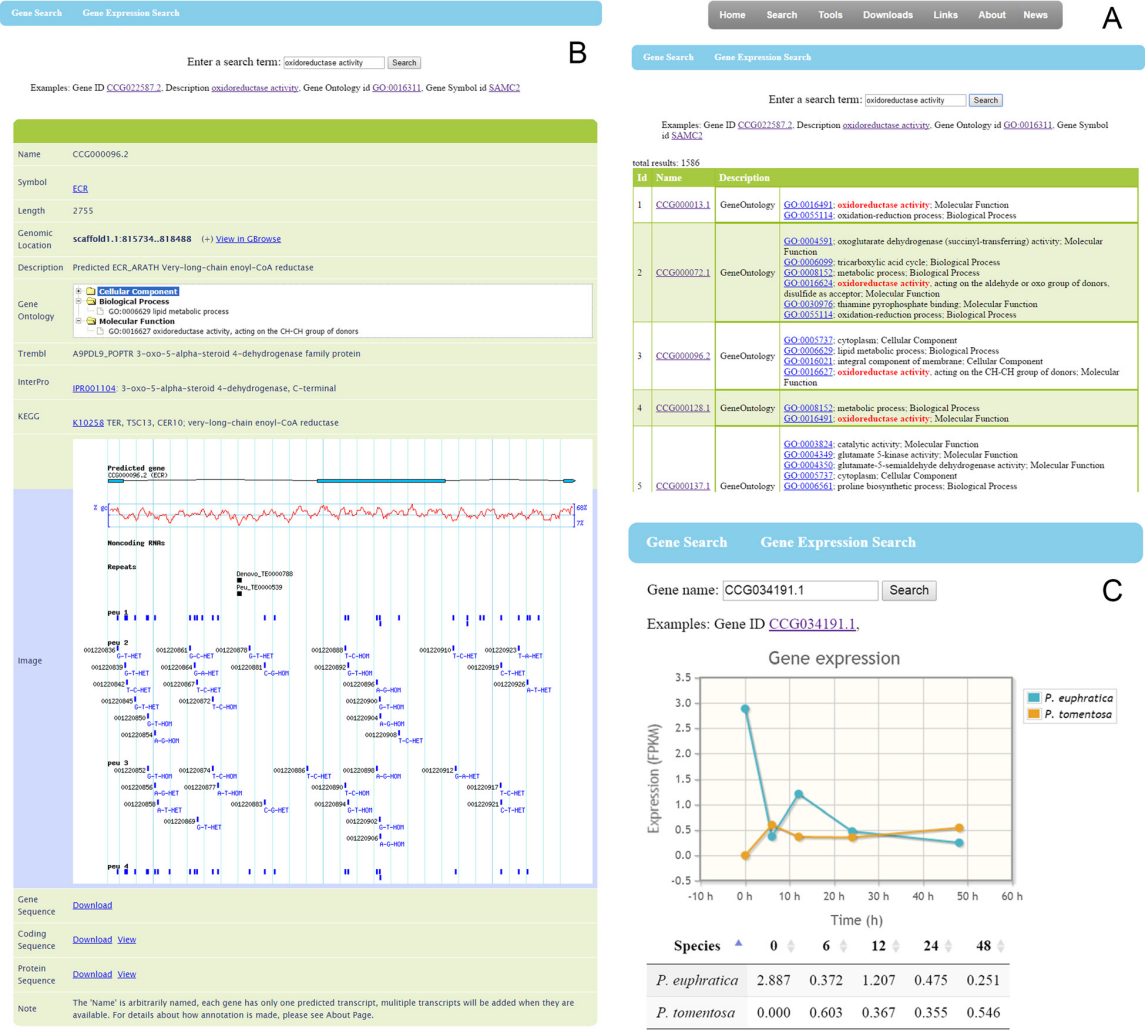
**Gene search. A**. Users can use Gene ID, keywords in description, Gene Ontology ID or Gene Symbol to find specific genes; **B**. Gene information page, which provides gene location and function annotation; **C**. Differentially expressed gene data between *P. euphratica* and *P. tomentosa* from the time-course profiling experiment.

The keyword search is a very useful function, which was developed to identify genes based on their names or annotations, such as “*MYB*” or “Betaine aldehyde dehydrogenase”. The detailed information about genes can be viewed by clicking on the gene's name or annotated identifier which links to relevant external website (Figure 2A). In the gene information page, users can view the annotated function, jump to GBrowse and download/view gene data (Figure 2B). This page also provides an overview of the gene's structure, genomic location and other related information. These annotations will be refreshed when gene models are updated. In the gene expression search page, the differentially expressed gene data between *P. euphratica* and *P. tomentosa* from the time-course profiling experiment is exhibited intuitively (Figure 2C).

### Genome browsing

**Figure 3:**
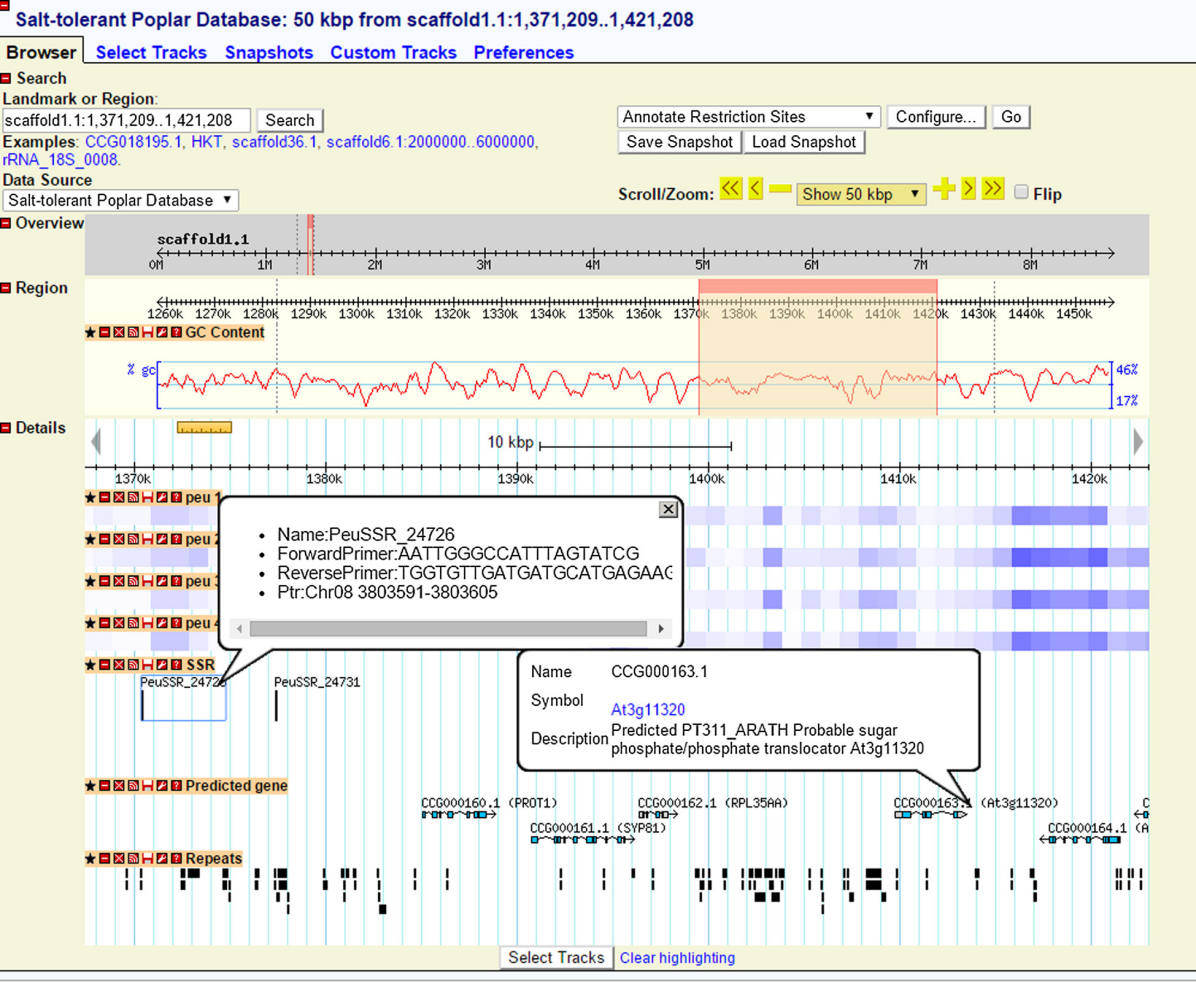
**Browsing genome using GBrowse.** Gene function description can be viewed by hovering the cursor over the gene name. When users click on a SSR name, a popup balloon will show the primer information and corresponding location in the *P. trichocarpa* genome.

The Generic Genome Browser (GBrowse; http://gmod.org/wiki/GBrowse) was used to visualize the genome of the *P. euphratica* [21]. Users can browse genes, repetitive elements, non-coding RNAs, SSRs and SNPs in different tracks, and zoom into any region of interest in the genome (Figure 3). By default, the repeats and SNPs are shown in density graph with darker color representing higher density. If the genome region is zoomed into certain ranges, summary mode will be turned off and detailed information of repeats and SNPs will be shown. When user hovers over a predicted gene or repeat element, a popup balloon will appear and show gene symbol, functional information or repeats family. By clicking on a SSR name, users will get the primer information and corresponding position in the *P. trichocarpa* genome. Clicking on a specific gene will redirect users to the corresponding gene information page, and users can return to the GBrowse by clicking on the “Tools” button within the navigation bar at the top of the page.

### Sequence similarity search

BLAST is one of the most useful tools for searching the STPD. It can be used to find similar sequences against scaffolds or genes within *P. euphratica* genome. In the page of blast searching result, each hit is linked to the GBrowse view of the sequence.

### Genome alignments browsing

**Figure 4:**
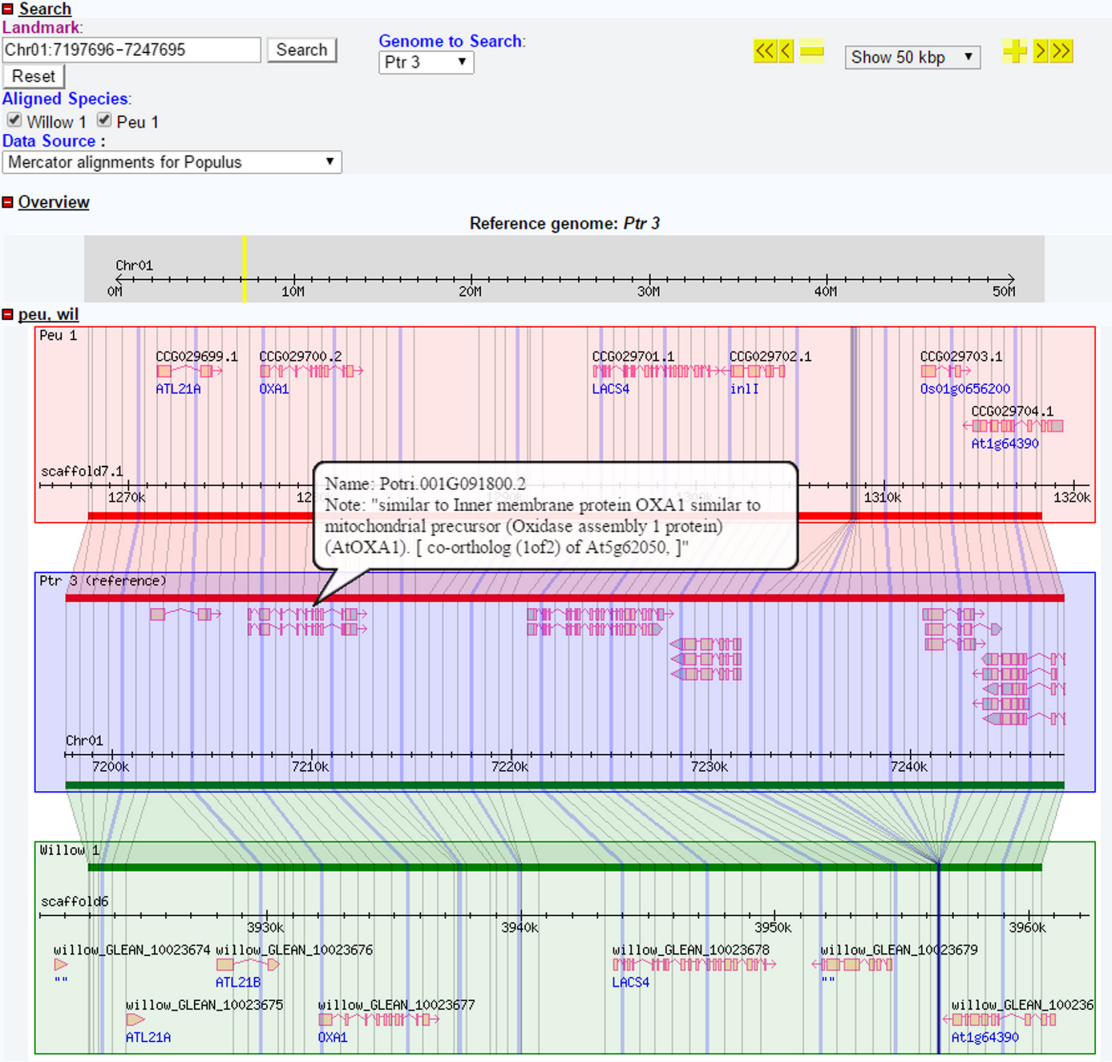
**Genome alignments among** ***P. euphratica*** **,** ***P. trichocarpa*** **and** ***S. suchowensis*** **.** Users can view any region of genome comparison by searching landmark or moving forward or backward using button in the right top corner.

The genome alignments can be viewed by the Generic Synteny Browser (GBrowse-syn), which uses the same software framework as GBrowse [22]. Therefore, gene symbols and functional information will also be showed in a popup balloon when user hovers over a specific gene (Figure 4). Users can copy “Genomic Location” from the gene information page, paste it into “Landmark” textbox, select “Peu 1” in “Genome to Search”, and then press search button to find the corresponding region in genome alignments.

### Data downloading

Users can download the deposited sequences from the STPD to perform local searches against the whole genome or further academic studies. The MD5 checksum is also available for checking file integration.

## Discussion

The STPD was created as a tool to facilitate the study of plant salt tolerance mechanism and poplar genomics. Compared with the existed poplar database (http://www.plantgdb.org/PtGDB/), our database provides access to a salinity tolerant poplar genome and its related genomic resources. It also offers comparative genomic data among *P. euphratica*, *P. trichocarpa* and *S. suchowensis* for researchers to better understand the adaptive evolutions of the closely related species. These data will promote the genetic dissection of stress-tolerant traits and accelerate the genetic improvement of cultivated poplars. The deposited sequences, related annotations and useful tools will allow users to find similar sequences in *Populus*, to browse genome features in the region of interest, and to retrieve data to further their interests in functional genomics, comparative genomics studies and molecular breeding. A number of research projects are currently in progress, including genome and transcriptome sequencing of *Populus pruinosa*, and genome re-sequencing of more than 200 individuals for *P. euphratica*, *P. pruinosa* and other related species. *P. pruinosa* is another salinity resistant poplar which is also distributed only in the desert regions of central Asia [23]. The genome-wide data getting from these studies will be integrated into the STPD as they become available.

## Conclusions

We have developed the STPD to support studies on tree adaptation to salt stress and poplar genomics. The database provides genome-wide datasets of salinity tolerant *Populus*, along with data mining and visualization tools for studying the salt resistance in poplars. New datasets will be incorporated in the future in order to expand the scope and to help scientists dissect the salt tolerance in *Populus* thoroughly*.* The STPD offers a valuable repository for scientific community to better study plant salt tolerance, comparative genomics, extreme environment adaptation and molecular breeding.

## Availability and requirements

**Database name:** STPD

**Database homepage:**http://me.lzu.edu.cn/stpd/

**Browser requirement:** JavaScript should be enabled; we recommend the use of the Chrome or Firefox web browsers for an optimal experience.

Datasets in the STPD are freely available for academic use. For all other uses, please contact Quanjun Hu, huquanjun@gmail.com.

## Abbreviations

BLAST: Basic local alignment search tool
BWA: Burrows wheeler aligner
GATK: Genome analysis tool kit
GBrowse: Generic genome browser
GBrowse-syn: Generic synteny browser kaas, kegg automatic annotation server
KEGG: Kyoto encyclopedia of genes and genomes
SNP: Single nucleotide polymorphism
SSR: Simple sequence repeat
STPD: Salinity Tolerant Poplar Database
TE: Transposable element
